# Analysis of clinical characteristics of 92 patients with paroxysmal nocturnal hemoglobinuria: A single institution experience in China

**DOI:** 10.1002/jcla.23008

**Published:** 2019-09-10

**Authors:** Rong Fu, Liyan Li, Lijuan Li, Hui Liu, Tian Zhang, Shaoxue Ding, Guojin Wang, Jia Song, Huaquan Wang, Limin Xing, Jing Guan, Zonghong Shao

**Affiliations:** ^1^ Department of Hematology Tianjin Medical University General Hospital Tianjin China

**Keywords:** glucocorticoid, paroxysmal nocturnal hemoglobinuria, renal injury, thrombosis

## Abstract

**Objectives:**

We performed a retrospective analysis to investigate the clinical characteristics and therapeutic strategies of Chinese paroxysmal nocturnal hemoglobinuria (PNH) patients, and assessed the efficacy and safety of glucocorticoid in PNH patients.

**Methods:**

The clinical data of 92 PNH cases in our hospital were analyzed, including clinical manifestation, laboratory examination, treatment efficacy, and survival.

**Results:**

The main clinical manifestations of these patients included hemoglobinuria, anemia, fatigue, dyspnea, headache, abdominal pain, and erectile dysfunction. Glucocorticoid is still the first‐line treatment for PNH patients to control hemolytic attack, and the short‐term remission rate (12 months) is 79.01% (64/81). Meanwhile, the overall survival (OS) of 10 years after diagnosis was estimated as 70.77% (46/65). Moreover, Cox proportional risk model for multivariate analysis showed that the increase in LDH multiple, thrombosis complications, and complicated with bone marrow failure were the independent adverse prognostic factors affecting the survival of PNH patients.

**Conclusion:**

Paroxysmal nocturnal hemoglobinuria patients in mainland China have various clinical features, while lower incidences of thrombosis and renal damage. Thrombosis and bone marrow failure are two complications with worse prognosis.

## 1 INTRODUCTION

Paroxysmal nocturnal hemoglobinuria (PNH) is a clonal hematopoietic stem cell disorder that manifests with hemolytic anemia, bone marrow failure, and thrombosis. Paroxysmal nocturnal hemoglobinuria originates from a multipotent hematopoietic stem cells that acquires a somatic mutation in a gene called phosphatidylinositol glycan anchor biosynthesis, class A (PIG‐A).^1^, ^2^The PIG‐A gene is required for the first step in glycosylphosphatidylinositol (GPI) anchor biosynthesis. Failure to synthesize GPI anchors leads to absence of all proteins that utilize GPI to attach to the plasma membrane.

The diagnosis of PNH is based on the detection of GPI‐anchored proteins (such as CD59, CD55) or the GPI‐anchor (flaer) by flow cytometry, which is more sensitive and replaced the Ham test and sucrose lysis tests.^3^ Significant advances have been reported in the detection of the GPI‐AP‐deficient cells, as well as in the pathophysiology of the disease. In treatment aspect, recombinant human anti‐complement C5 monoclonal antibodies, new complement inhibitors, and allogeneic hematopoietic stem cell transplantation have made great progress in the field of PNH therapy.^4^, ^5^, ^6^, ^7^, ^8^ China as a developing country, eculizumab is not yet available in China, and we have no experience with eculizumab, so the traditional treatment of PNH is aimed at protecting PNH clone, reducing complement attack and destruction, and alleviating hemolysis. Glucocorticoid is still the preferred first‐line drug for the treatment of PNH to control hemolysis attack. It has a definite effect. In order to better understand the clinical and laboratory features of PNH and to fully identify the development of complications and factors influencing survival in PNH in China, we herein undertook this retrospective study to review the patients with PNH admitted to our hospital from January 2005 to December 2015.

## 2 METHODS AND PATIENTS

### 2.1 Patients

Ninety‐two patients diagnosed as PNH from January 2005 to December 2015 in Tianjin Medical University General Hospital were enrolled in this retrospective study. It is worth mentioning that the 92 PNH patients enrolled in our study were all outpatients. Among them, patients with severe acute hemolytic episodes are treated in the income ward. For patients with mild illness, patients who did not have hemolysis were outpatient visits (including oral glucocorticoid therapy, PNH clone monitoring), and the income wards continued to be treated as soon as acute hemolysis or aggravation occurred. The clinical data of the patients were collected including gender, age, clinical classification, clinical manifestations, and laboratory findings. Paroxysmal nocturnal hemoglobinuria is classified into three different clinical forms: classic‐PNH, PNH in the setting of another bone marrow failure syndrome (PNH/AA, PNH/MDS), and subclinical PNH (non‐hemolytic PNH).^4^, ^9^ The PNH diagnostic criteria refer to Chinese expert consensus on the diagnosis and treatment of PNH (2014),^10^ and we also synthetically analyzed the results of the international PNH Working Group on PNH treatment.^11^, ^12^ The chronic kidney disease (CKD) classification was divided into five stages according to eGFR(mL/min/1.73 m^2^), which were as follows: grade 1 (eGFR > 90), grade 2 (eGFR60‐89), grade 3 (eGFR30‐59), grade 4 (eGFR15‐29), and grade 5 (eGFR < 15).

The research was in compliance of the declaration of Helsinki, and the protocol was approved by the ethical committee of Tianjin Medical University General Hospital. Consent for research and publication was obtained from participants and/or their immediate family if certain participants had passed away. In order to avoid losing clinical features, we collect the symptoms and signs of patients through the quality of life assessment questionnaire (FACIT Weakness Scale and EORTC QLQ‐C30 Questionnaire divided into questionnaires, general health status, and working status).

### 2.2 Treatment and therapeutic index

All patients received glucocorticoid treatment after diagnosis. The initial treatment included methylprednisolone intravenous infusion of 1 mg/kg/d and vitamin E (300 mg/day), and the dose of methylprednisolone may increase as appropriate if hemolytic crisis. Patients who had granulocytopenia (<1.5 × 10^9^) at admission were treated with granulocyte stimulating factor (2‐5 ug/kg d). And patients with severe anemia (Hb < 60 g/L) were treated with recombinant human erythropoietin injection (100‐150 IU/kg, subcutaneous injection, qod) besides erythrocyte transfusion. Some patients received component platelet transfusion if PLT < 10 × 10^9^/L. Thrombosis is one dangerous complication in PNH. For the 11 PNH patients with thrombotic complications, we used low molecular weight heparin anticoagulation therapy (enoxaparin sodium: 0.4 mg/d, 7‐14 days.) to anticoagulation. Except for two patients with bleeding tendency during the treatment, the other nine patients were treated with anticoagulation for 2 weeks.^13^, ^14^

Chinese hematology experts have formulated the criteria for the efficacy of glucocorticoids in the treatment of PNH,^10^ which are as follows: (a) full recovery: 1 years without hemoglobinuria episodes, no blood transfusion, blood routine examination (including reticulocytes) returned to normal; (b) complete remission: 1 years without hemoglobinuria, no blood transfusion, hemoglobin returned to normal; (c) partial remission: according to the classification of the condition before and after the observation, all the two levels of hemoglobin attack frequency, anemia severity and any progress in the condition of myelodysplastic were obviously improved; (d) improvement: there is progress in any classification of disease; (e) no response: no change or deterioration of the condition. (Note: during the observation period of more than 5 years, the word “recent” can be removed. The natural fluctuation of the disease must be ruled out when judging the therapeutic effect.)

According to clinical efficacy, the dosage of glucocorticoid was reduced to prednisone (0.5 mg/kg/d) if recent obvious progress. When the reticulocyte decreased and the hemoglobin remained at 100 g/L for more than 1 month, prednisone was slowly reduced to a minimum maintenance dose of 0.1‐0.2 mg/kg d for at least 3 months.

The median follow‐up was 59 months (18‐121). Overall survival (OS) was calculated from the time of first diagnosis of symptomatic PNH to the time of death. The end of the follow‐up was June 2017, or loss or death.

### 2.3 Statistical analysis

All data were analyzed by SPSS 21.0 statistical software. The classification variables were compared by Mann‐Whitney *U* test (two groups) or Kruskal‐Wallis test (three groups and above). Chi‐square test or Fisher exact test was used to test the difference. Kaplan‐Meier method was used to describe the distribution of survival status. Log‐rank test was used to analyze the prognosis of single factor, and Cox proportional regression model was used for multivariate analysis. *P* < .05 showed significant difference.

## 3 RESULTS

### 3.1 The characteristics of PNH patients

The clinical features and laboratory examinations of 92 PNH patients were showed in Table 1. In all the patients, 64 cases were classical PNH, 17 cases were PNH‐AA, and 11 cases were subclinical PNH. Clinical manifestations mainly include anemia (78.26%, 72/92), hemoglobinuria (54.35%, 50/92), fatigue (78.26%, 72/92), hemorrhage (13.04%, 12/92), dyspnea (42.39%, 39/92), abdominal pain (22.83%, 21/92), headache (27.17%, 25/92), thrombosis (11.96%, 11/92), dysphagia (6.52%, 6/92), erectile dysfunction (21.05%, 12/57), renal injury (14.13%, 13/92), impaired liver function (10.87%, 10/92), hepatomegaly (4.35%, 4/92), splenomegaly (5.43%, 5/92), and hepatosplenomegaly (1.09%, 1/92).

**Table 1:** The initial clinical characteristics of PNH patients

Characteristics	Patients n (%)
Total no. of patients	92
Gender M/F	57/35
Age median (range)	37 (18‐84)
Clinical classification n(%)	
Classical PNH	64 (69.57)
PNH‐AA	17 (18.48)
Subclinical PNH	11 (11.96)
Frequency of hemoglobinuria n(%)	
Frequent group	42 (46.15)
Sporadic group	31 (33.70)
Do not attack group	19 (20.65)
History of thrombosis n(%)	11 (11.96)
Parameters at baseline	
HGB (g/L)	67.89 ± 20.24
Ret (%)	8.48 ± 6.58
WBC (×10^9^/L)	4.38 ± 2.81
PLT (×10^9^/L)	73.11 + 57.80
TBIL (umol/L)	31.02 ± 19.64
DBIL (umol/L)	7.73 ± 4.91
LDH (U/L)	1377.09 ± 870.34
Cr (umol/L)	60.19 ± 27.47
Granulocyte CD59^−^ (%)	62.16 ± 25.15
Erythrocyte CD59^−^ (%)	35.71 ± 22.44
Flaer‐/CD14‐(%)	77.30 ± 21.28
Flaer‐/CD24‐(%)	82.65 ± 17.62
FHb > 50 mg/L	52 (64.20%)
Hp < 0.5 g/L	62 (76.54%)

Abbreviations: DBIL, direct bilirubin; HGB, hemoglobin; LDH, lactate dehydrogenase; N, number; PLT, platelet; PNH, paroxysmal nocturnal hemoglobinuria; RBC, red blood cell; RET, reticulocyte ratio; TBIL, total bilirubin; WBC, white blood cell.

### 3.2 Glucocorticoid treatment efficacy

**Figure 1:**
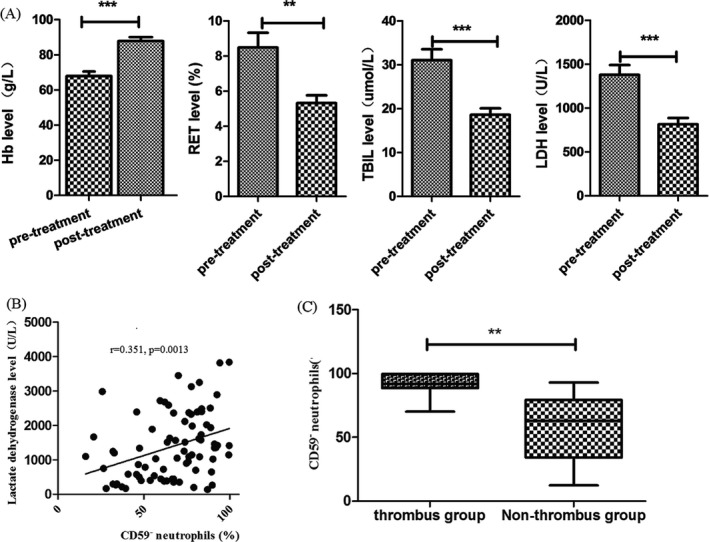
Glucocorticoid treatment efficacy in PNH patients. After 2‐4 wk treatment, the level of Hb increased significantly, while the levels of Ret, LDH, and TBIL were reduced, and the above hemolysis indicators were significantly different before and after treatment (A). The proportion of CD59‐negative neutrophils is positively related to the level of LDH in 81 PNH patients (*r* = .351, *P* = .0013) (B). The proportion of CD59‐negative neutrophils in 11 PNH patients with combined thrombosis is significantly higher than those without thrombosis (C) (*P* = .0028, ** represents *P *< .01, *** represents *P *< .001)

The treatment efficacy was analyzed in 81 cases, except 11 patients with subclinical PNH. The total response rate in 1 year was 79.01% (64/81), in which 8 (9.88%) cases were full recovery, 15 (18.52%) were complete remission, 28 (34.57%) were partial progress, 13 (16.05%) were improvement, and 17 (20.99%) were no response. After 2‐4 weeks of treatment, the level of Hb increased significantly (*P* = .001), while the levels of Ret, LDH, and TBIL were reduced (*P* values were .022, .000, and .001, respectively), indicating a good response (Figure 1A).

The total course of glucocorticoid treatment was 7.5 (2‐36) months. And the average duration of glucocorticoid treatment was (44.19 ± 19.96) days when Hb was stable over 100 g/L for 1 month. Moreover, 42 of 81 cases with severe anemia received red blood cell transfusion, and 18 patients with severe thrombocytopenia received platelet transfusion. After the treatment with glucocorticoid, 31 patients (73.81%) were independent of red blood cell transfusion, and the average time was (15.58 ± 9.72) days; seven patients (38.89%) were independent of the platelet transfusion, and the average time was (16.71 ± 4.27) days.

### 3.3 Overall survival

**Figure 2:**
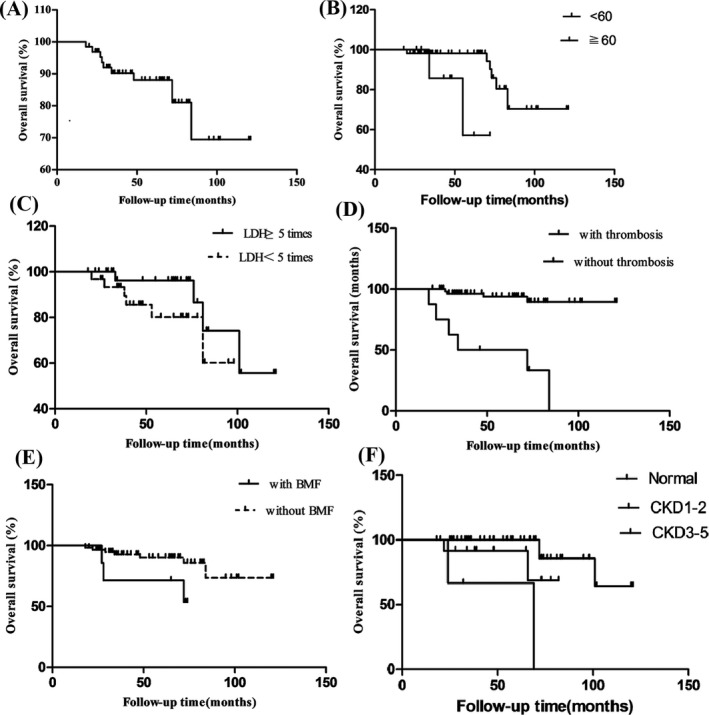
Survival rate in the cohort of the PNH patients. The overall survival (OS, %) in 10 y of all enrolled patients was showed (A), and then, the survival rates were observed according to the age (*P* = .026) (B), LDH increased times (*P* = .019) (C), combined with thrombosis (D) (*P* = .0001), combined with BMF (E) (*P* = .003), and the renal dysfunction (F) (*P* = .0001)

Of the 81 patients (except 11 patients with subclinical PNH), 16 patients were lost to follow‐up. The median treatment time was 7.5 (2‐36) months, and the OS of 10 years after diagnosis was estimated as 70.77%. We analyzed the overall survival of 65 PNH patients in terms of age, sex, PNH cloning ratio, LDH elevation multiples, thrombosis complications, and so on. The results showed that age, LDH elevation multiples, thrombosis complications, complicated with bone marrow failure, and renal dysfunction may be prognostic factors (Table 2, Figure 2A‐F). The above five factors were included in Cox proportional risk model for multivariate analysis, and the results showed that the increase in LDH multiple (*P* = .023), thrombosis complications (*P* = .0003), and complicated with bone marrow failure (*P* = .004) were the independent adverse prognostic factors affecting the survival of PNH patients.

**Table 2:** Clinical characteristics for univariate analysis of PNH patients

Variable	N (%)	10‐year OS	*P*
Number of patients	65	70.77%	
Age			
≧60	9 (13.85)	57.89%	.026
<60	56 (86.15)	71.43%	
Sex			
Male	37 (56.92)	72.84%	.653
Female	28 (43.08)	70.69%	
LDH increased times			
<5	34 (52.31)	71.75%	.019
≧5	31 (47.69)	41.12%	
CD59^−^ neutrophils			
≧50%	38 (58.46)	55.55%	.148
<50%	27 (41.54)	52.45%	
Combined thrombosis			
Yes	11 (16.92)	53 mo	.0001
No	54 (83.08)	88.92%	
Combined BMF			
Yes	17 (26.15)	50.45%	.003
No	48 (73.85)	71.75%	
Renal function			
Normal	50 (76.92)	72.09%	.0001
CKD1‐2	12 (18.46)	70.79%	
CKD3‐5	3 (4.62)	69 mo	

Abbreviations: BMF, bone marrow failure; CKD, chronic kidney disease; LDH, lactate dehydrogenase; OS, overall survival.

Twenty‐two (22/76) died after 10 years of follow‐up. The causes of death were thrombosis complications in nine patients, bleeding in six patients, and sudden death in seven patients (considering the relationship with anemic heart disease caused by long‐term anemia).

### 3.4 Adverse effects of glucocorticoid therapy

The most common adverse events during glucocorticoids were Iatrogenic Cushing's syndrome, infection, osteoporosis, and hyperglycemia. Seventeen patients had obvious iatrogenic Cushing's syndrome, including six cases of centripetal obesity, four cases of full‐moon face, and seven cases of skin purple lines. Upper respiratory tract infection occurred in two patients, one of whom developed pneumonia, one developed sepsis, one dental ulcer, one perianal infection, and one skin herpes. The symptoms of all patients with co‐infection were significantly improved after anti‐infection treatment without any sequelae. Bone mineral density examination showed that five patients had osteoporosis. Four patients had hyperglycemia, but after diet control and exercise blood glucose did not increase significantly and did not need to stop hormone therapy.

### 3.5 Complications

In our cohort, of the 81 patients (except 11 patients with subclinical PNH), 11 (13.58%) patients suffered from thrombosis, in which portal vein and mesenteric vein thrombosis in four patients, popliteal vein thrombosis in three patient, pulmonary embolism in two patient, and inferior vena cava, right common iliac vein thrombosis in three patient. Renal dysfunction or damage was a common complication in PNH patients as 16% of the study population exhibited stages 1‐3 CKD (Table 3). There is a significant difference in LDH between patients with normal renal function and CKD patients (*P* = .002), suggesting that hemolysis is an important cause of impaired renal function in patients with PNH. Approximately 13 (16.05%) patients had renal insufficiency comprising CKD stages 1‐3. About complications of infection, 33 (35.87%) patients were complicated with infection, among them, 21 cases of pulmonary infection (25.93%), six cases of upper respiratory tract infection (7.40%), three cases of urinary tract infection (3.70%), one cases of cholecystitis (1.23%), one cases of pancreatitis (1.23%), and one cases of skin and soft tissue infections (1.23%). The symptoms were improved after anti‐infection treatment.

**Table 3:** Renal function in all the PNH patients

CKD stages	eGFR (mL/min/1.73 m^2^)	N (%)	Granulocyte CD59‐(%)	LDH increased times
Normal		66 (81.48%)	51.60 ± 37.10	2.8 (0‐5.9)
One	>90, Albuminuria positive	10 (12.35%)	65.89 ± 47.71	4.7 (2.6‐6.8)
Two	60‐89, Albuminuria positive	2 (2.47%)	79.65 ± 36.74	6.9 (3.8‐10.4)
Three	30‐59	2 (2.47%)	77.56 ± 54.38	9.9 (5.7‐13.9)
Four	15‐29	1 (1.23%)	88.43 ± 49.52	10.54 (8.8‐13.1)
Five	<15	0		

Abbreviations: CKD, chronic kidney disease; eGFR, glomerular filtration rate; LDH, lactate dehydrogenase; N, number; PNH, Paroxysmal nocturnal hemoglobinuria.

We also observed the relationships between the size of PNH clones and complications. The proportion of CD59‐ neutrophil (NEU) in 81 patients was positively related to the level of LDH (*r* = .351, *P* = .0013) (Figure 1B). Because the increase in LDH level was an important sign of intravascular hemolysis, the size of PNH clone was related to hemolysis. The proportion of CD59‐ neutrophils in the patients with thrombosis (11 cases) was (88.71 ± 9.499)%, which was significantly higher than those without thrombosis (58.28 ± 24.27)% (*P* = .0028) (Figure 1C). The proportion of CD59‐ neutrophils was (63.30 ± 25.40)% in patients with impaired renal function (13 cases), which was no significant difference compared with the patients without renal impairment (68 cases) (57.78 ± 23.87)% (*P* = .4712).

## 4 DISCUSSION

PNH is a rare, life‐threatening, and debilitating clonal blood disorder caused by an acquired mutation in the phosphatidylinositol glycan (PIG)‐A gene. Clinically, PNH is characterized by a classical triad of acquired Coombs‐negative intravascular hemolytic anemia, thrombophilia, and various degrees of bone marrow failure.^15^, ^16^, ^17^ The resulting chronic intravascular hemolysis is the underlying cause of PNH morbidities and mortality. Our study showed that both sexes can be affected, and the ratio of male to female is 1.7:1. The median age of PNH patients at diagnosis was 37.4 years old, the male is slightly higher, the age span is very significant, and our case‐series study and previous reports revealed a slight preponderance of male in PNH.

The clinical manifestations included hemoglobinuria (54%), anemia (78%), hemorrhage (13%), pancytopenia (20.99%), and thrombus (12%). Though PNH was usually described as a intravascular hemolysis, PNH‐associated cytopenias were common in our study. Bone marrow failure is the PNH common clinical manifestations, occurs to some degree in all PNH patients, and, in its most extreme form, presents as immune‐mediated severe aplastic anemia. Although many patients showed a two‐line more blood cell reduce, but confirmed by bone marrow aspiration and biopsy, bad hyperplasia only 6.17%. We also evaluated the common symptoms of PNH patients and compared the differences between physician evaluation and patient self‐assessment. The results showed a significant difference in abdominal pain, headache, dysphagia, and erectile dysfunction. These differences remind us that we need to pay much attention to the common symptoms of PNH patients when they are interrogation, because patients often do not mention these symptoms voluntarily. Clinicians should ask patients voluntarily, so that they do not miss the data of these common symptoms. And we analyzed the correlation between the level of LDH and the ratio of CD59^−^ NEU, and the results showed that the size of PNH clones was positively correlated with hemolysis degree. The detection of PNH cloning provides the gold standard for diagnosis on the one hand and helps to predict clinical symptoms and complications on the other hand.^18^

The complications in the patients consist of recurrent infections, thrombotic events, hepatosplenomegaly, renal function damage, and abnormal coagulation function. In our study, recurrent infection was the main problem, which may be due to a significant proportion of the patients with bone marrow failure.

Later‐stage renal dysfunction or failure is a major cause of morbidity and mortality in PNH. Studies have reported that the five‐year mortality rate for PNH patients is about 35% and median survival time is about 10‐15 years, and kidney failure contributes to 8%‐18% of PNH‐related deaths.^19^ Clark 20 reported about 32% of PNH patients with renal failure (CKD3‐5). Previous reports showed that the mechanism of renal damage is considered to be persistent intravascular hemolysis, plasma‐free hemoglobin, and/or micro‐thrombus repeatedly acting on renal tissue. The large amount of free hemoglobin produced by hemolysis seriously consumes nitric oxide, which leads to the increase in renal artery resistance, the decrease in renal blood flow, and the impairment of renal function.^21^, ^22^ In our study of the 81 patients, one patient had been identified as specifically having chronic renal impairment in a review of their medical histories with later‐stage CKD (Stages 3‐5). Twelve of these 81 patients had been identified to have one or more major clinical kidney (MCK) events. And there is a significant difference in LDH between patients with normal renal function and CKD patients; therefore, it is speculated that the mechanism of PNH combined with renal damage may be closely related to intravascular hemolysis, and the specific mechanism of PNH combined with renal dysfunction needs further study in large samples.

The estimated OS of 10 years after diagnosis was 70.09% in our study, which was similar with 71% reported by Fujioka,^23^ and 75% by de Latour,^9^ but higher than 50% by Dacie and Lewis.^24^We compared the OS of 10 years after diagnosis from the following five aspects: the degree of LDH elevation, the degree of PNH clone increase, whether it was associated with thrombosis or bone marrow failure and the hemolytic attack frequency, the results showed that LDH increased times, combined with thrombosis and combined with bone marrow failure, all of them were adverse factors in the 10‐year survival rate reduction. The effect of CD59‐neutrophils proportions and hemolytic attack frequency on OS was not statistically significant, which might be related to the number of samples in each group we studied. This factor should be further analyzed after increasing the number of samples.

In acute hemolytic attack, glucocorticoid combined with hematopoietic growth factor, component support, and symptomatic support therapy can reduce acute hemolysis and stabilize the disease in most newly diagnosed patients. In our study, the treatment of total efficiency as high as 79.01%. But adverse reactions of long‐term high‐dose corticosteroid treatment to bring many side effects should be paid more attention. Therefore, how to effectively reduce PNH clonal abnormalities, maximum hemolysis control, as far as possible to reduce hormone dosage and prevent the hormone is the main problem to be urgently solved in our clinical work.

Thrombosis is one of the most feared complications and a major cause of death in patients with PNH.^25^, ^26^ Thrombus formation mechanism involves many aspects: vascular endothelial factor and high coagulation, deletion of GPI‐anchored protein‐urokinase type plasminogen activator receptor, role of free hemoglobin and depletion of nitric oxide and platelet dysfunction and increased platelet particle release.^15^, ^27^, ^28^, ^29^, ^30^ The incidence of thrombosis in patients with PNH in Europe is 28%‐40%, and the incidence of thrombosis in this study was 7.41%, which was significantly lower than that in western countries. The difference of the incidence of thrombosis in the East and West is not clear, whether it is related to the difference between the Asian race and the genetic gene of the western countries, it is worth further study.^31^ Among these patients in our study, eleven cases (13.58%) had complications associated with venous thrombosis. The incidence of thrombosis in our group of patients with abdominal vein thrombosis, lower extremity deep vein thrombosis, and cerebral thrombosis is more common, similar to foreign reports.

Eculizumab is a humanized monoclonal antibody (mAb) derived from the murine anti‐human C5 mAb; it binds to the complement component 5 (C5) and inhibits its further cleavage into C5a and C5b, disabling the progression to the terminal effector complement MAC. Many studies^4^, ^16^, ^32^, ^33^ have confirmed the effectiveness and safety of eculizumab: Eculizumab can reduce the demand for blood transfusion, improve anemia, alleviate the symptoms associated with chronic intravascular hemolysis (such as renal impairment), reduce pulmonary arterial pressure and serious thrombosis, and ultimately improve the quality of life and prolong the life period of the patients. Coversin, a new C5 complement inhibitor which can be injected subcutaneously, and phase II clinical trials are under way.^34^There is also research and development of oral C5 inhibitors.^35^These two formulations improve the inconvenience caused by intravenous injection of eculizumab. It is hoped that in the near future, eculizumab can be listed in China and benefit from PNH patients in China.

## 5 CONCLUSION

Paroxysmal nocturnal hemoglobinuria is a life‐threatening blood disorder in which patients are at risk for end‐organ damage and organ failure because of chronic hemolysis. Renal dysfunction or damage is a common and progressive medical complication in patients with PNH that also contributes to mortality in these patients. Our retrospective review of 92 PNH patients over a 10‐year period provided us a useful information, including the clinical manifestation, laboratory examination, treatment, complications, and prognostic factors influencing survival, which would help us to understand the pathogenesis of PNH.

## CONFLICT OF INTEREST

The authors declare that they have no conflict of interest.

## AUTHORS' CONTRIBUTIONS

Rong Fu and Zonghong Shao designed the research and revised the manuscript. Liyan Li, Hui Liu, and Lijuan Li performed the experiments, analyzed the data, and wrote the article. Tian Zhang, Shaoxue Ding, Guojin Wang, Jia Song, Huaquan Wang, Limin Xing, and Jing Guan contributed to the experimental work and the collection of patients' features. All authors read and approved the final manuscript.

## References

[1] Hillmen P , Lewis SM , Bessler M , Luzzatto L , Dacie JV . Natural history of paroxysmal nocturnal hemoglobinuria. N Engl J Med. 1995;333:1253‐1258. 7566002 10.1056/NEJM199511093331904

[2] Hill A , DeZern AE , Kinoshita T , et al. Paroxysmal nocturnal haemoglobinuria. Nat Rev Dis Primers. 2017;3:17028. 28516949 10.1038/nrdp.2017.28 PMC7879566

[3] Parker C , Omine M , Richards S , et al. Diagnosis and management of paroxysmal nocturnal hemoglobinuria. Blood. 2005;106:3699‐3709. 16051736 10.1182/blood-2005-04-1717 PMC1895106

[4] Parker CJ . Update on the diagnosis and management of paroxysmal nocturnal hemoglobinuria. Hematology Am Soc Hematol Educ Program. 2016;2016(1):208‐216. 27913482 10.1182/asheducation-2016.1.208 PMC6142517

[5] Yuan X , Gavriilaki E , Brodsky RA , et al. Small‐molecule factor D inhibitors selectively block the alternative pathway of complement in paroxysmal nocturnal hemoglobinuria and atypical hemolytic uremic syndrome. Haematologica. 2017;102(3):466‐475. 27810992 10.3324/haematol.2016.153312 PMC5394948

[6] Röth A , Rottinghaus ST , Hill A . Ravulizumab (ALXN1210) in patients with paroxysmal nocturnal hemoglobinuria: results of 2 phase 1b/2 studies. Blood Adv. 2018;2(17):2176‐2185. 30171081 10.1182/bloodadvances.2018020644 PMC6134221

[7] Kamranzadeh Fumani H , Zokaasadi M , Kasaeian A , et al. Allogeneic hematopoietic stem cell transplantation for paroxysmal nocturnal hemoglobinuria: a retrospective single‐center study. Hematol Oncol. 2017;35(4):935‐938. 27761934 10.1002/hon.2367

[8] Tominaga R , Katagiri T , Kataoka K , et al. Paroxysmal nocturnal hemoglobinuria induced by the occurrence of BCR‐ABL in a PIGA mutant hematopoietic progenitor cell. Leukemia. 2016;30(5):1208‐1210. 26437783 10.1038/leu.2015.268

[9] de Latour RP , Mary JY , Salanoubat C , et al. Paroxysmal nocturnal hemoglobinuria: natural history of disease subcategories. Blood. 2008;112:3099‐3106. 18535202 10.1182/blood-2008-01-133918

[10] Chinese Society of Hematology, Chinese Medical Association . Expert consensus of diagnosis and treatment of paroxysmal nocturnal hemoglobinuria. Zhonghua Xue Ye Xue Za Zhi. 2013;34:276‐279. 23683435 10.3760/cma.j.issn.0253-2727.2013.03.024

[11] Devalet B , Mullier F , Chatelain B , Dogné JM , Chatelain C . Pathophysiology, diagnosis, and treatment of paroxysmal nocturnal hemoglobinuria: a review. Eur J Haematol. 2015;95(3):190‐198. 25753400 10.1111/ejh.12543

[12] Chou WC , Huang WH , Wang MC , et al. Characteristics of Taiwanese patients of PNH in the international PNH registry. Thromb J. 2016;14(S1):39. 27766064 10.1186/s12959-016-0094-0 PMC5056488

[13] Griffin M , Munir T . Management of thrombosis in paroxysmal nocturnal hemoglobinuria: a clinician's guide. Ther Adv Hematol. 2017;8:119‐126. 28246555 10.1177/2040620716681748 PMC5305005

[14] Doutrelon C , Skopinski S , Boulon C , Constans J , Viallard JF , Peffault de Latour R . Paroxysmal nocturnal hemoglobinuria: an unknown cause of thrombosis. J Mal Vasc. 2015;40:384‐390. 26205796 10.1016/j.jmv.2015.06.006

[15] Hill A , Kelly RJ , Hillmen P . Thrombosis in paroxysmal nocturnal hemoglobinuria. Blood. 2013;121:4985‐4996. 23610373 10.1182/blood-2012-09-311381

[16] Luzzatto L , Risitano AM , Notaro R . Paroxysmal nocturnal hemoglobin and eculizumab. Haematologica. 2010;95:523‐526. 20378572 10.3324/haematol.2009.017848 PMC2857178

[17] Nishimura J , Kanakura Y , Ware RE , et al. Clinical course and flow cytometric analysis of paroxysmal nocturnal hemoglobinuria in the United States and Japan. Medicine. 2004;83:193‐207. 15118546 10.1097/01.md.0000126763.68170.46

[18] Kelly RJ , Hill A , Arnold LM , et al. Long‐term treatment with eculizumab in paroxysmal nocturnal hemoglobinuria: sustained efficacy and improved survival. Blood. 2011;117:6786‐6792. 21460245 10.1182/blood-2011-02-333997

[19] Socié G , Mary JY , de Gramont A , et al. Paroxysmal nocturnal haemoglobinuria: long‐term follow‐up and prognostic factors. French Society of Haematology. Lancet. 1996;348:573‐577. 8774569 10.1016/s0140-6736(95)12360-1

[20] Clark DA , Butler SA , Braren V , Hartmann RC , Jenkins DE Jr . The kidneys in paroxysmal nocturnal hemoglobinuria. Blood. 1981;57:83‐89. 7448417

[21] Hill A , Reid SA , Rother RP , et al. High definition contrast‐enhanced MR imaging in paroxysmal nocturnal hemoglobinuria (PNH) suggests a high frequency of subclinical thrombosis. Blood. 2006;108.

[22] Nath KA , Vercellotti GM , Grande JP , et al. Heme protein‐induced chronic renal inflammation: Suppressive effect of induced heme oxygenase‐1. Kidney Int. 2001;59:106‐117. 11135063 10.1046/j.1523-1755.2001.00471.x

[23] Fujioka S , Asai T . Prognostic features of paroxysmal nocturnal hemoglobinuria in Japan. Nihon Ketsueki Gakkai Zasshi. 1989;52:1386‐1394. 2629461

[24] Dacie JV , Lewis SM . Paroxysmal nocturnal hemoglobinuria: clinical manifestations, haematology, and nature of the disease. Ser Haematol. 1972;5:3‐23. 4565712

[25] Ziakas PD , Poulou LS , Pomoni A . Thrombosis in paroxysmal nocturnal hemoglobinuria at a glance: a clinical review. Curr Vasc Pharmacol. 2008;6:347‐353. 18855722 10.2174/157016108785909742

[26] Chen YL , Shou LH , Zhang ZX . Association of interleukin‐18 gene polymorphism and its protein expression with the lower extremity deep venous thrombosis in the Chinese Han population: a case‐control study. J Clin Lab Anal. 2018;32(4):e22345. 29105174 10.1002/jcla.22345 PMC6817231

[27] Van Bijnen ST , Van Heerde WL , Muus P . Mechanisms and clinical implications of thrombosis in paroxysmal nocturnal hemoglobinuria. J Thromb Haemost. 2012;10:1‐10. 22077430 10.1111/j.1538-7836.2011.04562.x

[28] Atfy M , Eissa M , Hossam E , Shabrawy DA . Role of urokinase plasminogen activator receptor (CD87) as a prognostic marker in acute myeloid leukemia. Med Oncol. 2012;29:2063‐2069. 21638078 10.1007/s12032-011-9993-x

[29] Fu R , Meng Y , Wang Y , et al. The dysfunction of platelets in Paroxysmal nocturnal hemoglobinuria. Thromb Res. 2016;12:50‐55. 10.1016/j.thromres.2016.07.012 27780113

[30] Ren P , Zhang J , Yu L , et al. Impact of different *Streptococcus pneumoniae* on the secretion of interleukin and adhesin from THP‐1 monocytes. J Clin Lab Anal. 2019;23:e22927. 10.1002/jcla.22927 PMC6757131 31231868

[31] Du Y , Long Z , Xie H , Zhuang J , Han B . The preliminary research in paroxysmal nocturnal hemoglobinuria with thrombosis. Zhonghua Xue Ye Xue Za Zhi. 2016;37:318‐323. 27093996 10.3760/cma.j.issn.0253-2727.2016.04.014 PMC7343087

[32] Choi CW , Jang JH , Kim JS , et al. Efficacy of eculizumab in paroxysmal nocturnal hemoglobinuria patients with or without aplastic anemia: prospective study of a Korean PNH cohort. Blood Res. 2017;52(3):207‐211. 29043236 10.5045/br.2017.52.3.207 PMC5641513

[33] Luzzatto L . Recent advances in the pathogenesis and treatment of paroxysmal nocturnal hemoglobinuria. F1000Res. 2016;23:5. 10.12688/f1000research.7288.1 PMC4765720 26962442

[34] Reddy YN , Siedlecki AM , Francis JM . Breaking down the complement system: a review and update on novel therapies. Curr Opin Nephrol Hypertens. 2017;26(2):123–128. 27977428 10.1097/MNH.0000000000000305

[35] Schubart A , Anderson K , Mainolfi N , et al. Small molecule factor B inhibitor for the treatment of complement-mediated diseases. Proc Natl Acad Sci USA. 2019;116(16):7926–7931. 30926668 10.1073/pnas.1820892116 PMC6475383

